# SG-APSIC1176: Laboratory-acquired COVID-19 during the SARS-CoV-2 ο (omicron) pandemic wave at a tertiary-care hospital in Korea

**DOI:** 10.1017/ash.2023.16

**Published:** 2023-03-16

**Authors:** Mi-Na Kim, Joonsang Yu, Hun Hong

**Affiliations:** Asan Medical Center, Seoul, Republic of Korea; Asan Medical Center University of Ulsan College of Medicine, Seoul, Republic of Korea; Asan Medical Center University of Ulsan College of Medicine, Seoul, Republic of Korea

## Abstract

**Objectives:** Laboratory-acquired infection (LAI) of SARS-CoV is well known, but MERS-CoV or SARS-CoV-2 LAI has not yet been reported. Beginning last November, COVID-19 cases increased among laboratory staff at our 2,700-bed tertiary-care hospital. A 7-day home-quarantine policy for healthcare workers when household members were confirmed with SARS-COV-2 was lifted February 28. We investigated LAI and its risk factors. **Methods:** From March 21 to 25, all confirmed cases of COVID-19 among 176 laboratory staff were surveyed with questionnaire to collect the following data: symptom onset and period, SARS-CoV-2 PCR–positive sample date, age, sex, infection in household members, close contact with COVID-19 confirmed staff, work type, work unit, possibility of LAI and LAI risk factors. **Results:** In total, 54 laboratory staff (30.1%) were confirmed with SARS-CoV-2 infection; first 1 person on November 28 and 1 person on November 30, 2021, then 13 in February 2022 and 39 later in 2022. Overall, 22 cases had previously infected household members, and 9 cases suspected that they had had hospital contact with an infected patients through phlebotomy or bedside tests. In total, 25 cases of possible LAI mainly occurred in clusters of 3, 6, or 7 people through person-to-person transmission of a coworker who had an infected family member. The remaining 9 cases, including 1 sample receptionist, 2 urine analysis technicians, and 6 SARS-CoV-2 PCR test staff, may have been infected through an infected sample. However, person-to-person transmission was still possible because most shared a changing room and lounge in the same work unit. **Conclusions:** The most important cause of LAI is person-to-person transmission between coworkers; therefore, home quarantine is an effective measure to prevent LAI when a household member is infected wish SARS-CoV-2. Handling of infected specimens may be the second most common cause of LAI.

